# Supplementary material for: Molecular Epidemiology and Diversity of SARS-CoV-2 in Ethiopia, 2020–2022
Source: Genes (Basel). 2023 Mar 13;14(3):705. doi: 10.3390/genes14030705 (PMC10047986; doi:10.3390/genes14030705)
Supplement: Supplementary file 1 [file genes-14-00705-s001.zip › genes-2222985-supplementary.pdf]

Supplementary Materials:

Table S1. Summary of the COVID-19 timeline and major events following the first confirmed cases in Ethiopia

Timeline	Major events
December 2019/January 2020	Wuhan City, Hubei Province, China, was reported to the WHO on 31 December 2019. Wuhan, China, was reported in January 2020
27 January 2020	Prior to the WHO's declaration of a global pandemic, the federal democratic republic of Ethiopia Council of Ministers 'activated' a National Public Health Emergency Preparedness Centre, EOC and began preparations to deal with a potential outbreak of Covid-19
30 January 2020	the Director-General of the WHO declared COVID-19 a Public Health Emergency of International Concern
7 February 2020	Ethiopia set up the national Influenza and Arboviruses reference laboratory as the first COVID-19 testing laboratory in the country with support from WHO and ACDC.
March 11, 2020-	WHO declared COVID-19 a Pandemic
March 13, 2020,	On 12 March 2020, a week after entering the country from Burkina Faso, a 48-year-old Japanese national presented himself at a public health center in the capital city, Addis Ababa, and was diagnosed as having COVID-19. Since Ethiopia did not have the capacity to test for COVID-19 at the onset. Thus, samples were initially transported for testing to the National Institute for Communicable Diseases (NICD) Laboratory in South Africa
15 March 2020	The second confirmed case from Ethiopian nationalities (three additional cases) of COVID-19 was reported
20 March 2020	Anyone entering the country/international travelers/ should stay in quarantine for up to 14 days at their costs
23 March 2020	Ethiopia closed all land borders and deployed security forces to halt the movement of people along the borders (National lockdown….)
24 March 2020	• banned all public gatherings and sports events<br>• The closure of bars and clubs<br>• all federal employees were to work from home, except those designated by each ministry and federal agency as essential workers
05 April 2020	The First confirmed COVID-19-related death
8 April 2020	A state of emergency was declared giving authorities across-the-board powers to battle the disease
06 March 2021 **after a year of pathogen occurrence*	The first genome sequence of SARS-CoV-2 sampled from Ethiopia was submitted to the GISAID database
13March, 2021	First vaccination started

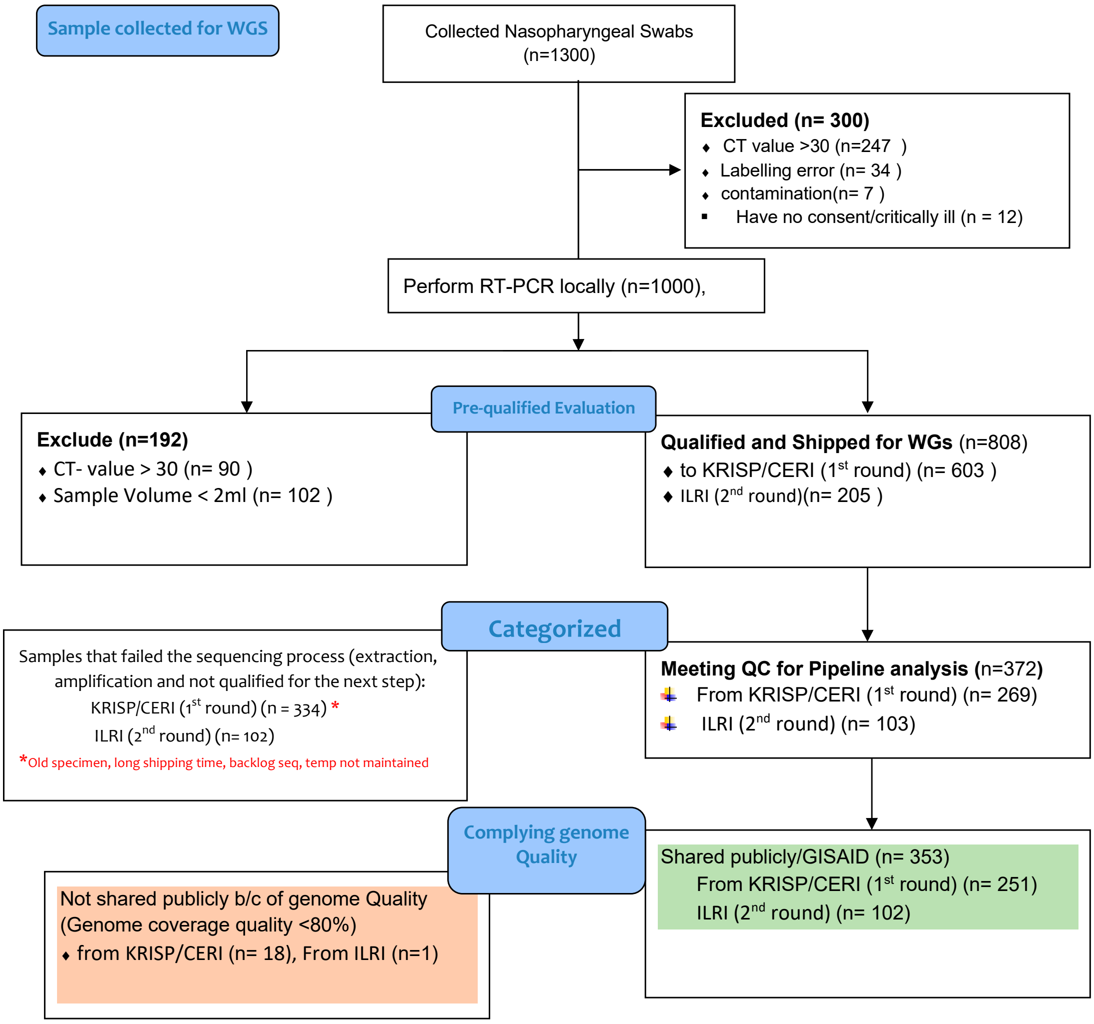

Figure S1: Consortium Diagram of Sampling for Molecular Epidemiology and Diversity of SARS-CoV-2 in Ethiopia

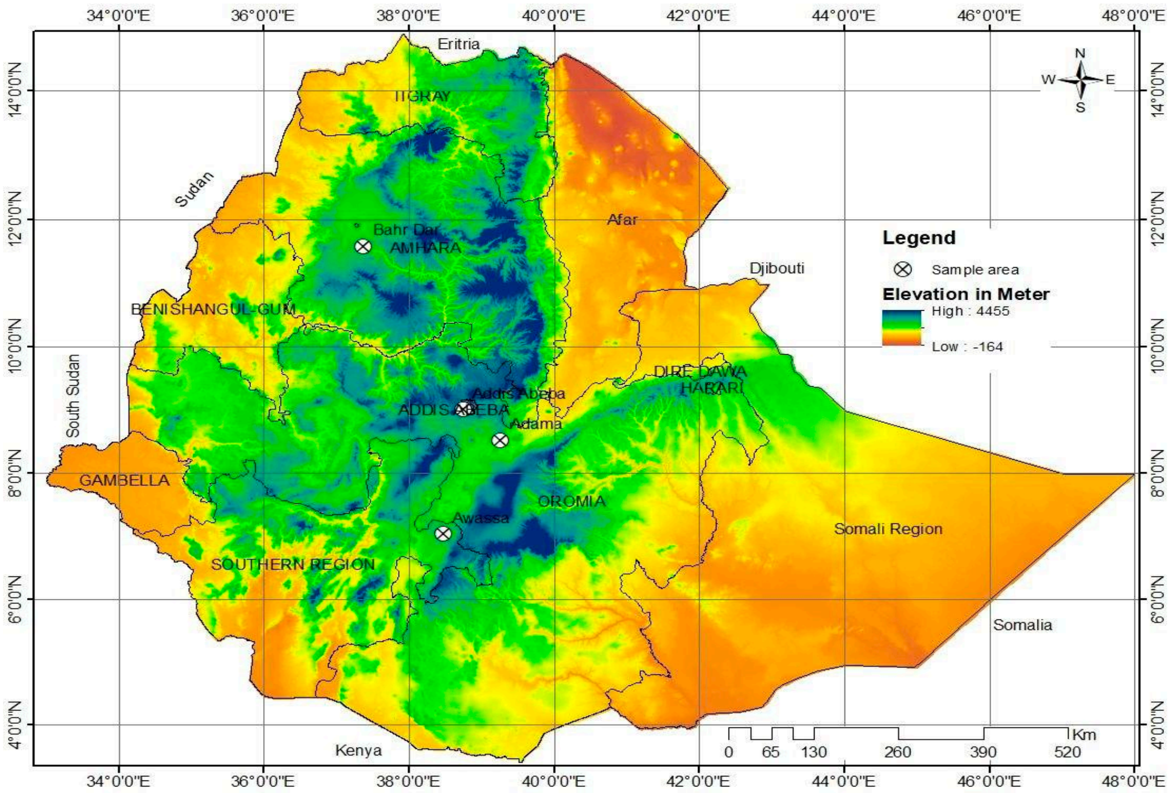

Figure S2: Sampling area for Molecular Epidemiology and Diversity of SARS-CoV-2 in Ethiopia

Table S2: Sequence proportion of the regional government

Name of Region	The proportion of sequences (%)
Addis Ababa	80%
Oromia	8%
Amhara	5%
SNNPR and Sidama	5%
Others	2%

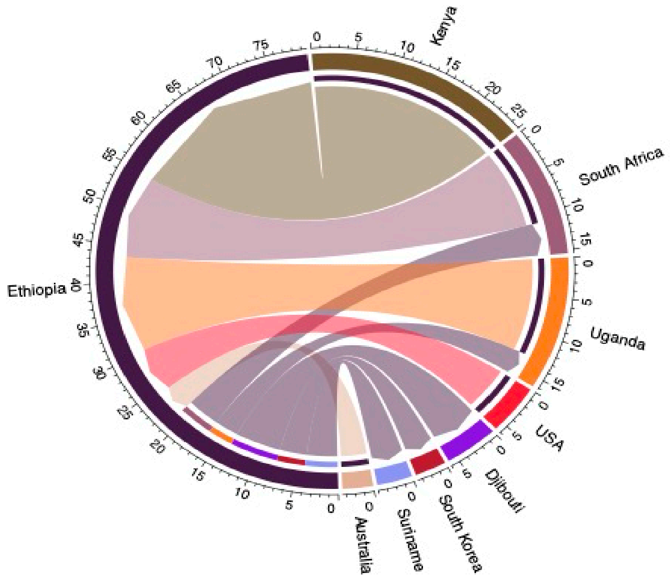

Figure S3: Import-Export Analyses of SARS-CoV-2 lineages in Ethiopia using chord plot. Chord plot showing the exchange of SARS-CoV-2 viruses between Ethiopia and other countries as inferred by import and export analysis for the period between June 2020 and February 2022. The flat end from Ethiopia denotes viral exports from Ethiopia, while the pointed ends toward Ethiopia denote viral imports into Ethiopia. The scale represents mean import/exports as inferred against 10 replicates

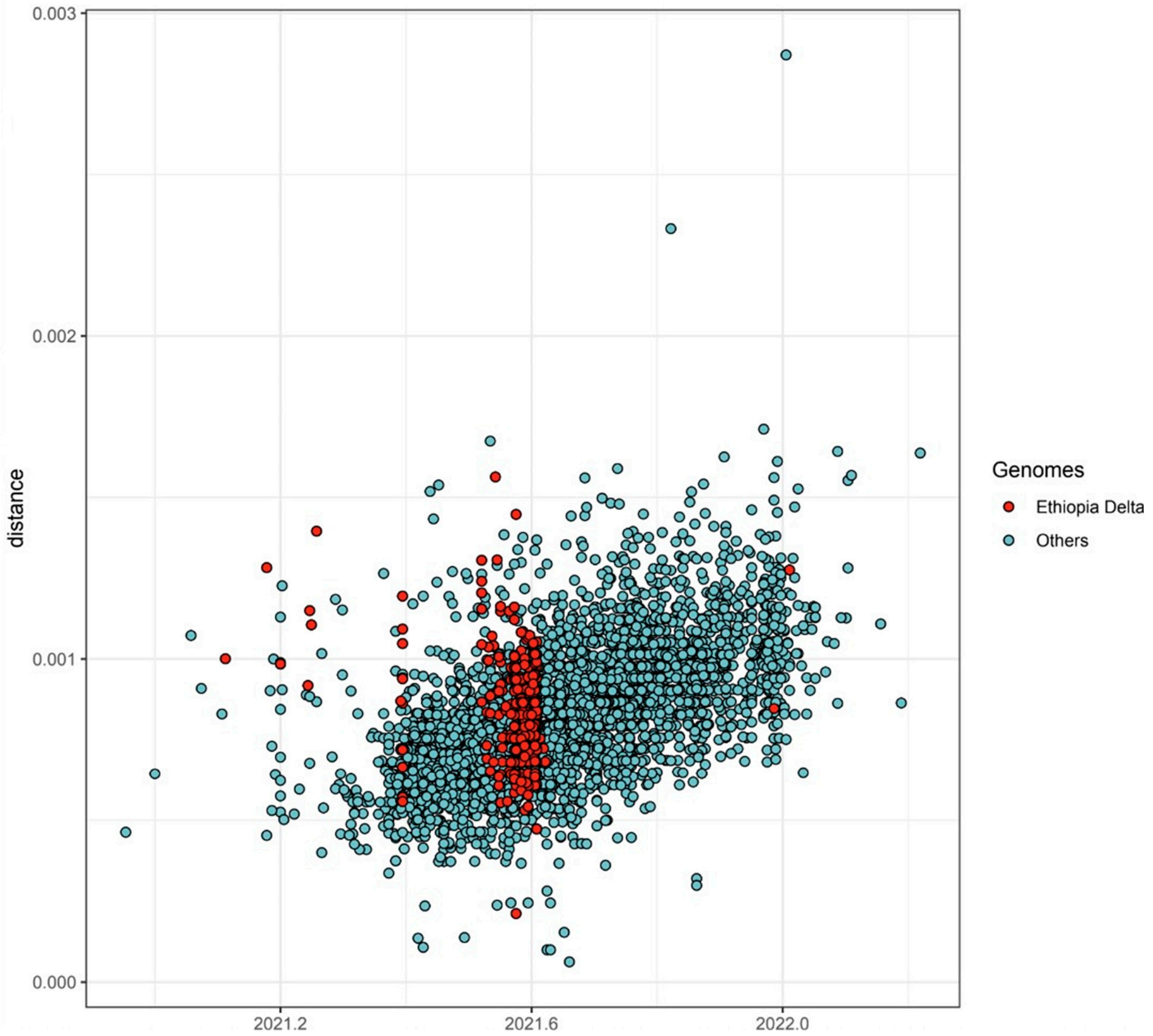

Figure S4: SARS-CoV-2 Delta VOC sub tree molecular clock, Ethiopia.

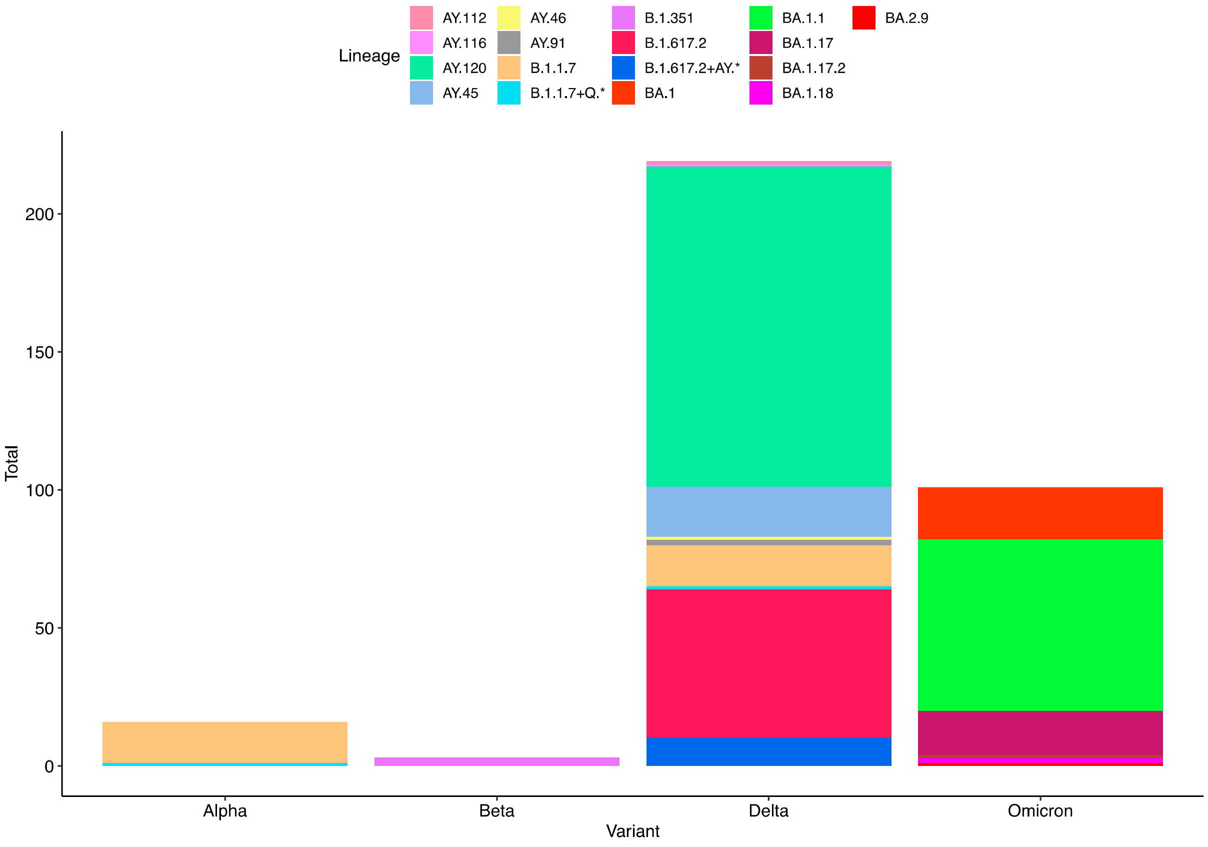

Figure S5: Major SARS-CoV-2 lineages circulating in Ethiopia between June 2020 and February 2022
